# REMOTE Ischemic Perconditioning Among Acute Ischemic Stroke Patients in Catalonia: REMOTE-CAT PROJECT

**DOI:** 10.3389/fneur.2020.569696

**Published:** 2020-09-25

**Authors:** Francisco Purroy, Gloria Arque, Gerard Mauri, Cristina García-Vázquez, Mikel Vicente-Pascual, Cristina Pereira, Daniel Vazquez-Justes, Coral Torres-Querol, Ana Vena, Sònia Abilleira, Pere Cardona, Carles Forné, Xavier Jiménez-Fàbrega, Jorge Pagola, Manuel Portero-Otin, Ana Rodríguez-Campello, Àlex Rovira, Joan Martí-Fàbregas

**Affiliations:** ^1^Stroke Unit, Department of Neurology, Hospital Universitari Arnau de Vilanova de Lleida, Lleida, Spain; ^2^Clinical Neurosciences Group, Institut de Recerca Biomèdica de Lleida (IRBLleida), Universitat de Lleida, Lleida, Spain; ^3^Stroke Programme, Agency for Health Quality and Assessment of Catalonia, CIBER Epidemiología y Salud Pública (CIBERESP), Barcelona, Spain; ^4^Stroke Unit, Hospital de Bellvitge, Hospitalet de Llobregat, Spain; ^5^Department of Basic Medical Sciences, Universitat de Lleida, Lleida, Spain; ^6^Servei d'Emergències Mèdiques, Hospitalet de Llobregat, Spain; ^7^Stroke Unit, Neurology Department, Vall d'Hebron Hospital, Barcelona, Spain; ^8^Department of Experimental Medicine, NUTREN-Nutrigenomics, Biomedical Institut de Recerca Biomèdica de Lleida (IRBLleida), Universitat de Lleida, Lleida, Spain; ^9^Neurovascular Research Group, Neurology Department, Institut Hospital del Mar d'Investigacions Mèdiques-Hospital del Mar, Departament de Medicina, Universitat Autònoma de Barcelona, Barcelona, Spain; ^10^Section of Neuroradiology and MRI Unit, Department of Radiology, Hospital Universitari Vall d'Hebron, Universitat Autònoma de Barcelona, Barcelona, Spain; ^11^Stroke Unit, Hospital de Sant Pau, Barcelona, Spain

**Keywords:** ischemic stroke, remote ischemic perconditioning (rPerC), neuroprotection, infarct size (IS), metabolomics (OMICS)

## Abstract

**Rationale:** Remote ischemic perconditioning during cerebral ischemia (RIPerC) refers to the application of brief episodes of transient limb ischemia commonly to a limb, it represents a new safe, simple and low-cost paradigm in neuroprotection.

**Aim and/or Hypothesis:** To evaluate the effects of RIPerC on acute ischemic stroke (AIS) patients, applied in the ambulance, to improve functional outcomes compared with standard of care.

**Sample Size Estimates:** A sample size of 286 patients in each arm achieves 80% power to detect treatment differences of 14% in the outcome, using a two-sided binomial test at significance level of 0.05, assuming that 40% of the control patients will experience good outcome and an initial misdiagnosis rate of 29%.

**Methods and Design:** We aim to conduct a multicentre study of pre-hospital RIPerC application in AIS patients. A total of 572 adult patients diagnosed of suspected clinical stroke within 8 h of symptom onset and clinical deficit >0 according to prehospital rapid arterial occlusion evaluation (RACE) scale score will be randomized, in blocks of size 4, to RIPerC or sham. Patients will be stratified by RACE score scale. RIPerC will be started in the ambulance before hospital admission and continued in the hospital if necessary. It will consist of five cycles of electronic tourniquet inflation and deflation (5 min each). The cuff pressure for RIPerC will be 200 mmHg during inflation. Sham will only simulate vibration of the device.

**Study Outcome(s):** The primary outcome will be the difference in the proportion of patients with good outcomes as defined by a mRS score of 2 or less at 90 days. Secondary outcomes to be monitored will include early neurological improvement rate, treatment related serious adverse event rates, size of the infarct volume, symptomatic intracranial hemorrhage, metabolomic and lipidomic response to RIPerC and Neuropsychological evaluation at 90 days.

**Discussion:** Neuroprotective therapies could not only increase the benefits of available reperfusion therapies among AIS patients but also provide an option for patients who are not candidates for these treatments. REMOTE-CAT will investigate the clinical benefit of RIC as a new neuroprotective strategy in AIS.

**Clinical Trial Registration:** www.ClinicalTrials.gov, identifier: NCT03375762.

## Introduction and Rationale

Stroke is one of the leading causes of death worldwide and the main cause of disability (1). Currently, the only therapies for acute ischemic stroke (AIS) patients are the administration of rt-PA (2) and/or endovascular treatment (3). Unfortunately, many patients cannot benefit from these therapies due to their contraindications or evolution time. Neuroprotective therapies could not only increase the benefits of available reperfusion therapies but also provide an option for patients who are not candidates for these treatments (4). However, most neuroprotection trials have so far failed to demonstrate their efficacy in AIS patients, despite promising results in animal studies (4). Remote ischemic perconditioning (RIPerC) represents a new paradigm in neuroprotection (5). It potential upregulates endogenous defense systems to achieve ischemic tolerance in brain ischemia (6). It consists of brief episodes of transient limb ischemia. According to studies in coronary ischemia, RIPerC during the ischemic event is safe, feasible, and related to a decrease in myocardial injury (7). However, there is limited data about the clinical utility of RIPerC in AIS patients. Only four randomized clinical trials (RCTs) have been completed and published (8–11). All of them demonstrated that RIC is safe and feasible in AIS. One has been conducted to test RIPerC in a prehospital setting in AIS patients and as an adjunct treatment with intravenous alteplase (11). Two other small-size studies were only designed to evaluate the safety and feasibility of RIC in AIS patients recruited within 24 h of onset of symptoms (8) and in alteplase treated patients (9). The last and the largest study included 188 patients with confirmed carotid ischemic stroke within 6 h of symptoms onset (10). None of them demonstrated a significant clinical effect or a significant effect on brain infarction volume growth.

We aim to conduct a multicentre study of pre-hospital RIPerC in AIS patients applied within 8 h of stroke onset. Our hypothesis is that RIPerC would be safe and would induce endogenous neuroprotective phenomena associated with good outcomes in AIS patients treated with revascularization therapies or not.

## Methods

### Design

REMOTE-CAT is a prospective randomized controlled multicentre clinical trial that follows CONSORT statement (12). The study will be performed in accordance with the standards of good clinical practice (International committee on Harmonization of E6 Guideline for Good Clinical Practice) and the latest revision of the Declaration of Helsinki. The study has been approved by the Ethics Committee on Clinical Research of the Hospital Universitari Arnau de Vilanova of Lleida (approval code 1744). All patients will provide written informed consent. The protocol is registered in ClinicalTrials.gov identifier is NCT03375762.

### Patient Population

**Figure 1:**
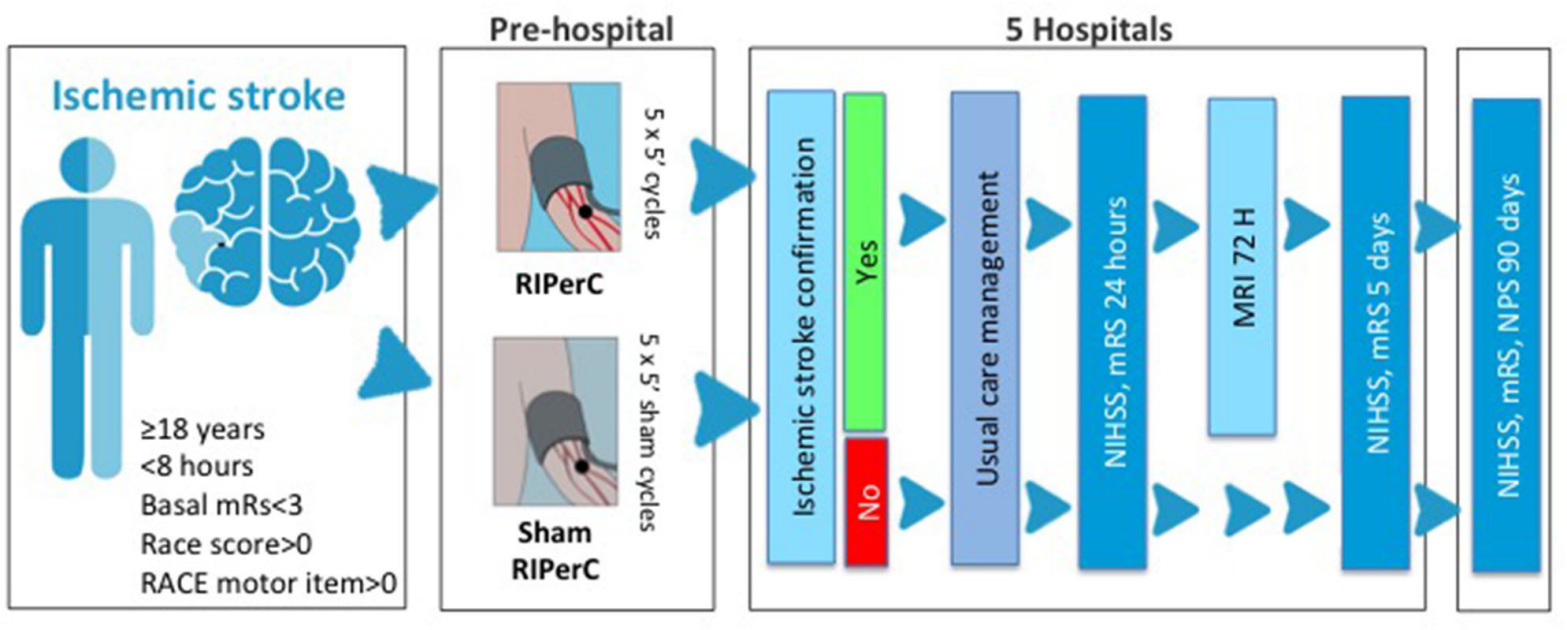
Graphical representation of REMOTE-CAT clinical trial procedures. After Stroke Code activation in the ambulance, remote ischemic perconditioning (RIPerC) is applied by an automatic device during transportion to the nearest Stroke care center. Usual medical care will be performed in the stroke center.

Inclusion and exclusion criteria are detailed in Table 1. Summarizing, REMOTE-CAT focuses on patients with suspected acute stroke identified in the pre-hospital setting by emergency medical services (EMS). The EMS is a public company responsible for urgent prehospital care including Code Stroke (CS) patients. We will include consecutive adult subjects (age ≥18 years old) with CS activation will be included. CS activation criteria include neurologic impairment suggestive of acute stroke according to FAST criteria (13), time from symptom onset of <8 h and previous functional independence (modified Rankin Scale, mRS ≤ 2). Patients should have at least motor impairment (Figure 1). Baseline assessments and study procedures are reported in Table 2.

**Table 1:** Inclusion and exclusion criteria.

**Inclusion criteria**
• Age above 18 years old • Suspected clinical stroke within 8 h of onset of neurological symptoms • Stroke code (SC) activation • Independent in daily living before the acute onset of symptoms (mRs ≤ 2) • RACE score>0 and RACE motor score>0 • Written informed consent (patient or legal representative)
**Exclusion criteria**
• Unknown onset of symptoms • Coma (GCS <8) • Malignancy or significant comorbidity thought to limit life expectancy to <6 months • Pregnancy • Participation in other clinical trial related with a research medical product/device

*RACE, The rapid arterial occlusion evaluation scale; mRs, Modified Rankin scale; GCS, Glasgow Coma Scale*.

**Table 2:** Study procedures for eligible patients with AIS.

**Time points**	**Prehospital**	**Admission**	**24 h**	**3 days**	**5 days**	**3 months**
Enrollment						
Eligibility screen	✓					
Patient/family information	✓	✓				
Acute waiver of consent	✓					
Randomization	✓					
RACE scale	✓					
Blood pressure	✓	✓^‡^				
Informed consent		✓				
AIS confirmation		✓^*^			✓^*^	
Demographics and medical history		✓				
Reperfusion therapies		✓				
Intervention						
RIC/Sham application	✓					
Complications related to RIC		✓	✓		✓	✓
Assessment						
NIHSS		✓	✓		✓	✓
Modified Rankin scale		✓			✓	✓
Neuroimaging		✓	✓^#^			
MRI				✓		
Blood biomarkers		✓		✓	✓	
Stroke etiology					✓	
Quality of life						✓
Neuropsychological evaluation						✓
Safety measurement						
Intrahospital complications					✓	✓
SICH			✓		✓	

RACE, rapid arterial occlusion evaluation; AIS, acute ischemic stroke; RIC, remote ischemic conditioning; MRI, magnetic resonance imaging; NIHSS, National Institutes of Health Stroke Scale.

‡ Blood pressure will be reported at the end of RIC/Sham application and 1 h after.

* Patients with intracranial hemorrhage will complete the 90 days follow-up. Patients without evidence of brain ischemia will be followed until discharge and through registries.

# *Patients treated with intravenous rtPA and/or EVT will undergo neuroimaging at 24 h*.

### Randomization

First, patients will be stratified using the rapid arterial occlusion evaluation (RACE) scale score (14) and then, they will be randomly allocated in blocks of size four, to either receive remote ischemic conditioning (RIPerC group) or sham in the ambulance. An on-call physician not involved in the study will perform the randomization using a computer program located in a web server.

### Intervention

RIPerC will consist of automatically delivered five cycles of electronic tourniquet inflation to the upper non-paretic limb, each lasting 5 min and separated by 5 min of cuff deflation. The cuff will be inflated to 200 mmHg and it will be applied to the opposite arm to the one experiencing motor and/or sensory deficit in order to reduce the risk of phlebitis and to maintain the somatosensory stimuli. RIPerC will be initiated by the ambulance staff during transportation and it will be finished for all patients in the ambulance or during Hospital admission. All patients in both groups will be treated according to conventional care procedures following international guidelines. Thus, mechanical thrombectomy and intravenous fibrinolysis will be allowed. Revascularization therapies will not be delayed due to the study. The non-interventionist group will use a sham device. It will simulate vibration of the device but no inflation will be performed. Discomfort and complications related to RIPerC will be recorded.

### Primary Outcome

The primary outcome will be the difference in the proportion of patients with good outcomes as defined by a mRS score of 2 or less at 90 days.

### Secondary Outcomes

The secondary outcomes will be: (1) a decrease in the National Institutes of Health Stroke Scale (NIHSS) score greater or equal than 4 between baseline and day one, 5 ± 1 days and 90 ± 7 days; (2) a mRS score of 2 or less at 5 ± 1 days; (3) the rate of serious adverse events related to the intervention; (4) the rate of symptomatic intracerebral hemorrhage (SICH) defined by the Safe Implementation of Thrombolysis in Stroke Monitoring Study protocol at 24–36 hours (15); (5) acute infarct volume; (6) metabolomic and lipidomic response to RIC; and, (7) neuropsychological evaluation of cognitive and affective domains.

In all eligible patients, a brain MRI will be performed within 3–4 days of the onset of symptoms, including the following sequences: (1) transverse T2-FLAIR; (2) transverse T2^*^-weighted gradient-echo; (3) transverse diffusion-weighted (DWI) single-shot echo-planar spin-echo; and, (4) axial 3D time-of-flight MR angiography (through the circle of Willis). All participating centers will follow the same protocol. A neuroradiologist blinded to clinical features and intervention will review the MRI images. Infarct volume will be defined as the hyperintense area on the initial isotropic DWI acquired with a b value of 1,000 sec/mm2.

In addition, we will use metabolomic and lipidomic analyses to define a panel of serum biomarkers accurately related to RIC phenomenon. For these purposes, in 100 patients (50 sham and 50 RIPerC), blood samples for further determination of metabolomics and lipidomics will be drawn at arrival to hospital, at days 3 and 5 as previously performed (16, 17). As we did not have preliminary data and no metabolites have yet been defined we will performed a non-targeted metabolomics and lipidomic profiling in order to identify differential molecules found in the intervention group.

The neuropsychological evaluation will include Montreal cognitive assessment, trail making test part a and b, the Wechsler adult intelligence scale, the free and cued selective reminding test, the apathy evaluation scale and the Rey complex figure test. We also performed the Health-related quality of life assessment questionnaire (EQ-5D) at 90 days.

### Data Monitoring Body

An independent data safety monitoring board (DSMB) will look after the safety of the study. It will ensure that the rate of SICH and serious adverse events is similar in the two groups. An interim analysis will be performed after the inclusion of the first 100 patients for early stopping due to safety reasons. Moreover, the DSMB could recommend stopping the study for safety reasons at any moment and make recommendations to the Executive Committee regarding efficacy, quality and feasibility of the study.

### Sample Size Estimates

A sample size of 280 subjects in each arm achieves 80% power to detect treatment differences of 14% between the intervention and the control groups, using a two-sided binomial test at significance level of 0.05, assuming that 40% of the control patients will experience good outcome defined by a mRS score of 2 or less at 90 days, and allowing a misdiagnosis rate of 29% (15% of haemorrhagic strokes and 14% of mimic stroke conditions). Two extra interim analyses will be performed at 33 and 67% of recruitment on the primary outcome. Early stopping is planned if large differences between the study groups are observed in order to reduce study participants' exposure to the inferior study arm and saving time and resources. Since repeated significance testing on accumulating data will be performed, adjustment of the usual hypothesis testing procedure to maintain the overall significance level of 0.05 will be done by using the flexible type I error spending function (18, 19). Due to the interim analyses, the sample size must be increased by an inflation factor of 1.02, resulting in 572 patients (286 per group).

### Statistical Analyses

Included patients will be described with respect to demographic and clinical characteristics, according to the study arm. Continuous variables will be summarized using means and standard deviations for normally distributed data; or median and 25–75% percentiles for non-normally distributed data. Normal distribution will be assessed by means of the Shapiro–Wilks test, rejecting normality when *p* <0.05. Categorical data will be summarized using counts and percentages. Comparisons will be performed by means of the Pearson's chi-squared test for categorical variables; the *t*-test for normally distributed data; and the Mann–Whitney *U*-test for non-normally distributed data.

Primary analysis will be performed by means of the binomial test. If the study groups are unbalanced, the primary outcome will be compared using a logistic regression model that will include the variables exhibiting baseline differences as covariates.

Secondary outcomes will be compared using the most appropriate test according to the distribution of the data. As with the primary analysis, secondary outcomes and safety outcome analyses will be conducted using multivariable generalized linear models with suitable links.

The analysis will be performed on the intention-to-treat set and will be repeated on the per-protocol set as a sensitivity analysis.

### Stratified Analysis

All objectives will be assessed in the following stratified analyses: (i) by sex; (ii) depending on whether patients have undergone thrombectomy; (iii) depending on whether patients have undergone thrombectomy and treated with rtPA; (iv) whether patients have undergone thrombectomy or treated with rtPA; and (v) number of cycles of inflation and deflation finished to evaluate a possible dose-response effect.

### Handling Missing Data

If there are missing data, they will be reported for each variable and missingness mechanism will be explored. Missing values could depend on other observed data. We will consider these missing values as missing at random (MAR). If there is no correlation between the missing values and other observed data (i.e., the Little's test is not statistically significant, *p* > 0.05) missing values will be considered missing completely at random (MCAR) (20). If missing values are MAR, a series of multiple imputations by chained equations will be performed and the Rubin's rules will be used to combine variable estimates and standard errors (21). If missing values are MCAR, complete case analysis will be performed.

### Current Status of the Trial

The study started recruitment in August 2019 in one Hospital, and the estimated completion date is August 2022. At 1st August 2020: 76 patients have been recruited.

### Study Organization and Funding

FP is the coordinating investigator of the study, which is funded by a grant from the Spanish National Ministry of Heath—PI17/01725.

## Discussion

RIPerC emerges as an interesting neuroprotective strategy (5). Our study improves the previous limited experience in humans (8, 11). It includes all AIS with symptom onset within 8 h and not only intravenous alteplase treated patients (11). According to the animal model, it is effective when applied both alone and in combination with revascularization therapies (22, 23). Although few studies have been published about the effect of RIC in AIS, some important issues have been learned (8–11). As in Hougaard's trial (11) and in most of the trials involving patients with myocardial infarction (7, 24) it is applied it in the ambulance, as soon as possible, in order to induce the maximum effect. This action seems to be safe although the definitive diagnosis will not be established until the arrival at the hospital. To date, no serious adverse effects have been reported in RIC studies (5, 7–11, 25). The recent RCT published by Pico et al. failed to demonstrate an effect of RIC in the final infarction size in AIS. One explanation of their neutral results was that the treatment with RIC was performed too late during or after the receipt of reperfusion therapies (10). In addition, we will increase the number of cycles to five. Most RIC trials use the four-cycle protocol (7, 11) due to tradition. The ischemic conditioning phenomena was first demonstrated using this protocol in an animal model of myocardial infarction (26). Recent studies in animal models address the need to increase the number of cycles in order to optimize the efficacy of RIPerC (27). Some other recent successes in remote ischemic preconditioning (28) and chronic postconditioning (29) in AIS patients have used the 5-cycle protocol. Increasing the duration of the cycle to 10 min does not offer any further protection (27). Although the quantity of muscle mass affects the efficacy of the intervention, we decided to perform the RIPerC on an upper arm rather than on a leg for safety reasons as up to one in four AIS patients have silent peripheral arterial disease defined by a low ankle-brachial index (30). One of the main shortcomings of the previously mentioned Hougaard's trial (11) was that fewer than one out of three patients complete the four cycles of limb ischemia. We will therefore use an automatic device. To avoid misdiagnosis only subjects with RACE score of >0 and RACE motor items >0 will be included. Finally, according to the stroke treatment academic industry roundtable (STAIR) recommendations a clinical endpoint would clearly evaluate the utility of applying RIPerC in AIS patients.

## Summary and Conclusions

RIPerC represents a new paradigm in neuroprotection with limited data in AIS patients. According to previous preclinical and clinical studies of acute ischemia, a clinical RIPerC trial should include both candidates and non-candidates for reperfusion therapies. As the RIPerC effect decreases with time, RIPerC should be started during the transfer of stroke code patients. The size of the trial should be large enough to detect differences in clinical outcomes and not only neuroimaging endpoints. Finally, the RIC device should be automatic to not only ensure that patients finish all of the programmed cycles but also to interfere as little as possible with the work of paramedics during the transfer and of nurses and physicians during the admission.

## Ethics Statement

The studies involving human participants were reviewed and approved by the Ethics Committee on Clinical Research of the Hospital Universitari Arnau de Vilanova de Lleida. The patients/participants provided their written informed consent to participate in this study.

## Author Contributions

FP conceived the study and wrote the paper. FP, GM, CG-V, MV-P, DV-J, and GA were involved in protocol development and study conduct. GA, CF, and SA substantively revised the manuscript. All authors read and approved the final manuscript.

## Conflict of Interest

The authors declare that the research was conducted in the absence of any commercial or financial relationships that could be construed as a potential conflict of interest.

## References

[1] Mozaffarian D Benjamin EJ Go AS Arnett DK Blaha MJ Cushman M American heart association statistics and stroke statistics, heart disease and stroke statistics-2016 update: a report from the American heart association. Circulation. (2016) 133:e38–360. 10.1161/CIR.0000000000000350 26673558

[2] Demaerschalk BM Kleindorfer DO Adeoye OM Demchuk AM Fugate JE Grotta JC. American heart association stroke, council on and prevention, scientific rationale for the inclusion and exclusion criteria for intravenous alteplase in acute ischemic stroke: a statement for healthcare professionals from the American heart association/American stroke association. Stroke. (2016) 47:581–641. 10.1161/STR.0000000000000086 26696642

[3] Saver JL Goyal M van der Lugt A Menon BK Majoie CB Dippel DW. Time to treatment with endovascular thrombectomy and outcomes from ischemic stroke: a meta-analysis. JAMA. (2016) 316:1279–88. 10.1001/jama.2016.13647 27673305

[4] Chamorro A Dirnagl U Urra X Planas AM. Neuroprotection in acute stroke: targeting excitotoxicity, oxidative and nitrosative stress, and inflammation. Lancet Neurol. (2016) 15:869–81. 10.1016/S1474-4422(16)00114-9 27180033

[5] Hess DC Blauenfeldt RA Andersen G Hougaard KD Hoda MN Ding Y. Remote ischaemic conditioning-a new paradigm of self-protection in the brain. Nat Rev Neurol. (2015) 11:698–710. 10.1038/nrneurol.2015.223 26585977

[6] Purroy F Garcia C Mauri G Pereira C Torres C Vazquez-Justes D. Induced neuroprotection by remote ischemic perconditioning as a new paradigm in ischemic stroke at the acute phase, a systematic review. BMC Neurol. (2020) 20:266. 10.1186/s12883-020-01836-8 32615939 PMC7330956

[7] Man C Gong D Zhou Y Fan Y. Meta-analysis of remote ischemic conditioning in patients with acute myocardial infarction. Sci Rep. (2017) 7:43529. 10.1038/srep43529 28272470 PMC5341091

[8] England TJ Hedstrom A O'Sullivan S Donnelly R Barrett DA Sarmad S. RECAST (Remote ischemic conditioning after stroke trial): a pilot randomized placebo controlled phase II trial in acute ischemic stroke. Stroke. (2017) 48:1412–15. 10.1161/STROKEAHA.116.016429 28265014

[9] Che R Zhao W Ma Q Jiang F Wu L Yu Z. rt-PA with remote ischemic postconditioning for acute ischemic stroke. Ann Clin Transl Neurol. (2019) 6:364–72. 10.1002/acn3.713 30847368 PMC6389851

[10] Pico F Lapergue B Ferrigno M Rosso C Meseguer E Chadenat ML. Effect of in-hospital remote ischemic perconditioning on brain infarction growth and clinical outcomes in patients with acute ischemic stroke: the RESCUE BRAIN randomized clinical trial. JAMA Neurol. (2020) 77:1–11. 10.1001/jamaneurol.2020.0326 32227157 PMC7105950

[11] Hougaard KD Hjort N Zeidler D Sørensen L Nørgaard A Hansen TM. Remote ischemic perconditioning as an adjunct therapy to thrombolysis in patients with acute ischemic stroke: a randomized trial. Stroke. (2014) 45:159–67. 10.1161/STROKEAHA.113.001346 24203849

[12] Schulz KF Altman DG Moher D Group C. CONSORT 2010 statement: updated guidelines for reporting parallel group randomised trials. BMJ. (2010) 340:c332. 10.1136/bmj.c332 20332509 PMC2844940

[13] Harbison J Hossain O Jenkinson D Davis J Louw SJ Ford GA. Diagnostic accuracy of stroke referrals from primary care, emergency room physicians, and ambulance staff using the face arm speech test. Stroke. (2003) 34:71–6. 10.1161/01.STR.0000044170.46643.5E 12511753

[14] Perez de la Ossa N Carrera D Gorchs M Querol M Millan M Gomis M. Design and validation of a prehospital stroke scale to predict large arterial occlusion: the rapid arterial occlusion evaluation scale. Stroke. (2014) 45:87–91. 10.1161/STROKEAHA.113.003071 24281224

[15] Wahlgren N Ahmed N Davalos A Ford GA Grond M Hacke W. Thrombolysis with alteplase for acute ischaemic stroke in the safe implementation of thrombolysis in stroke-monitoring study (SITS-MOST): an observational study. Lancet. (2007) 369:275–82. 10.1016/S0140-6736(07)60149-4 17258667

[16] Purroy F Cambray S Mauri-Capdevila G Jove M Sanahuja J Farre J Metabolomics predicts neuroimaging characteristics of transient ischemic attack patients. EBioMed. (2016) 3964:30514–X. 10.1016/j.ebiom.2016.11.010 PMC5161417 27843094

[17] Jove M Mauri-Capdevila G Suarez I Cambray S Sanahuja J Quilez A. Metabolomics predicts stroke recurrence after transient ischemic attack. Neurology. (2015) 84:36–45. 10.1212/WNL.0000000000001093 25471397 PMC4336096

[18] Kim K DeMets DL. Confidence intervals following group sequential tests in clinical trials. Biometrics. (1987) 43:857–64. 10.2307/2531539 3427170

[19] Lan KKG DeMets DL Discrete sequential boundaries for clinical trials. Biometrika. (1983) 70:659–63. 10.1093/biomet/70.3.659

[20] Jakobsen JC Gluud C Wetterslev J Winkel P. When and how should multiple imputation be used for handling missing data in randomised clinical trials - a practical guide with flowcharts. BMC Med Res Methodol. (2017) 17:162. 10.1186/s12874-017-0442-1 29207961 PMC5717805

[21] White IR Royston P Wood AM. Multiple imputation using chained equations: issues and guidance for practice. Stat Med. (2011) 30:377–99. 10.1002/sim.4067 21225900

[22] Hoda MN Siddiqui S Herberg S Periyasamy-Thandavan S Bhatia K Hafez SS. Remote ischemic perconditioning is effective alone and in combination with intravenous tissue-type plasminogen activator in murine model of embolic stroke. Stroke. (2012) 43:2794–9. 10.1161/STROKEAHA.112.660373 22910893 PMC3740528

[23] Hahn CD Manlhiot C Schmidt MR Nielsen TT Redington AN. Remote ischemic per-conditioning: a novel therapy for acute stroke? Stroke. (2011) 42:2960–2. 10.1161/STROKEAHA.111.622340 21836089

[24] McLeod SL Iansavichene A Cheskes S. Remote ischemic perconditioning to reduce reperfusion injury during acute st-segment-elevation myocardial infarction: a systematic review and meta-analysis. J Am Heart Assoc. (2017) 6:e005522. 10.1161/JAHA.117.005522 28515120 PMC5524098

[25] Zhao W Che R Li S Ren C Li C Wu C. Remote ischemic conditioning for acute stroke patients treated with thrombectomy. Ann Clin Transl Neurol. (2018) 5:850–6. 10.1002/acn3.588 30009202 PMC6043766

[26] Murry CE Jennings RB Reimer KA. Preconditioning with ischemia: a delay of lethal cell injury in ischemic myocardium. Circulation. (1986) 74:1124–36. 10.1161/01.CIR.74.5.1124 3769170

[27] Johnsen J Pryds K Salman R Lofgren B Kristiansen SB Botker HE. The remote ischemic preconditioning algorithm: effect of number of cycles, cycle duration and effector organ mass on efficacy of protection. Basic Res Cardiol. (2016) 111:10. 10.1007/s00395-016-0529-6 26768477

[28] Zhao W Meng R Ma C Hou B Jiao L Zhu F. Safety and efficacy of remote ischemic preconditioning in patients with severe carotid artery stenosis before carotid artery stenting: a proof-of-concept, randomized controlled trial. Circulation. (2017) 135:1325–35. 10.1161/CIRCULATIONAHA.116.024807 28174194 PMC5802341

[29] Meng R Asmaro K Meng L Liu Y Ma C Xi C. Upper limb ischemic preconditioning prevents recurrent stroke in intracranial arterial stenosis. Neurology. (2012) 79:1853–61. 10.1212/WNL.0b013e318271f76a 23035060

[30] Purroy F Coll B Oró M Setó E Piñol-Ripoll G Plana A. Predictive value of ankle brachial index in patients with acute ischaemic stroke. Eur J Neurol. (2010) 17:602–6. 10.1111/j.1468-1331.2009.02874.x 19968705

